# Monitoring of antimicrobial usage among adult bovines in dairy herds of Punjab, India: A quantitative analysis of pattern and frequency

**DOI:** 10.3389/fvets.2023.1089307

**Published:** 2023-03-30

**Authors:** Deepthi Vijay, Jasbir Singh Bedi, Pankaj Dhaka, Randhir Singh, Jaswinder Singh, Anil Kumar Arora, Jatinder Paul Singh Gill

**Affiliations:** ^1^Centre for One Health, College of Veterinary Science, Guru Angad Dev Veterinary and Animal Sciences University, Ludhiana, India; ^2^Department of Veterinary and Animal Husbandry Extension Education, College of Veterinary Science, Guru Angad Dev Veterinary and Animal Sciences University, Ludhiana, India; ^3^Department of Veterinary Microbiology, College of Veterinary Science, Guru Angad Dev Veterinary and Animal Sciences University, Ludhiana, India

**Keywords:** antimicrobial usage, BIN method, bovines, dairy, milk

## Abstract

The present study aimed to evaluate the antimicrobial usage (AMU) pattern in dairy herds of Punjab, India. The on-farm quantification of AMU in adult bovine animals by the manual collection of empty drug containers (“bin method”) along with the records of the treatment was carried out in 38 dairy farms involving 1010 adult bovines for 1 year from July 2020 to June 2021. The farm owners were asked to record the antibiotic treatments as well as to deposit empty antibiotic packaging/vials into the provided bins placed at the farms. A total of 14 different antibiotic agents in 265 commercial antibiotic products were administered to the dairy herds during the study. A total of 179 (67.55%) administered products contained antimicrobials of “critical importance” as per the World Health Organization (WHO). Mastitis (54.72%), followed by the treatment of fever (19.62%), reproductive problems (15.47%), and diarrhea (3.40%) accounted for the majority of drugs administered in the herds during the study period. The most commonly used antibiotics were enrofloxacin (89.47% herds; 21.51% products), followed by ceftriaxone (50% herds; 12.83% products), amoxicillin (50% herds; 12.83% products), oxytetracycline (55.26% herds; 11.70% products), and procaine penicillin (47.37% herds; 12.83% products). The highest quantity of AMU [in terms of antimicrobial drug use rate (ADUR)] was observed for ceftiofur, followed by ceftriaxone, procaine benzyl penicillin ceftizoxime, enrofloxacin, cefoperazone, amoxicillin and ampicillin. A total of 125 (47.17%) products contained “highest priority critically important antimicrobials” (HPCIA) and 54 (20.37%) products contained “high priority critically important antimicrobials”. In terms of overall number of animal daily doses (nADD), the highest priority critically important antimicrobials (HPCIA) of the WHO such as third-generation cephalosporins and quinolones, respectively accounted for 44.64 and 22.35% of the total antibiotic use in the herds. The bin method offers an alternative to monitoring AMU as a more accessible approach for recording the actual consumption of antimicrobials. The present study, to the best of our knowledge, is the first of its kind to provide an overview of the qualitative and quantitative estimate of AMU among adult bovines from India.

## 1. Introduction

There is a projected rapid rise in the global human population to 9.8 billion by 2050, where almost half of the world's population growth is expected in developing countries (1). The population growth is generating a huge demand for livestock products, particularly for milk in developing countries, which is predicted to increase by 62% by 2050 (2–4). This increased demand for livestock products has promoted intensive livestock farming with high antimicrobial use for therapeutics, prophylaxis, as well as for growth promotion, which may lead to the emergence of antimicrobial resistance (AMR) (5–7). By 2030, the livestock industry is projected to account for 70% of the total antimicrobial use (AMU) globally, and antibiotic use in the animal husbandry sector of India has been predicted to double by this period (8).

As notified by the World Health Organization (WHO), the judicious use of antimicrobials especially “Critically Important Antimicrobials (CIAs) for human medicine” is crucial for AMR mitigation as well as for public health security (9). The categorization of the antimicrobials into “critically important”, “highly important” and “important” in the WHO list of “Critically Important Antimicrobials for human medicine” (WHO CIA list) aims to ensure the prudent use of medically important antimicrobials for humans in the animal husbandry sector (10). In line with the various global action plans on combating AMR, the Government of India have also launched the “National Action Plan on Antimicrobial Resistance” (NAP-AMR) in 2017 (11), with one of the aims to optimize the use of antimicrobials in animals by restricting the use of antibiotics which are critically important for humans. However, there are implementation gaps in the NAP-AMR, as the field-level regulatory measures are still in the initial stages (12).

India stands fifth in terms of veterinary antimicrobial consumption in food animals measured in terms of veterinary antimicrobial sales data (13). However, there could be considerable bias in estimating the AMU based on sales data of veterinary antibiotics as it gives limited information on the number and species of animals treated, the condition of their use or the duration of treatment (14). Thereby, it is crucial to have a proper assessment of antibiotic usage in the animal husbandry sector at the regional as well as national levels, which can serve as a basis for the risk assessment of AMR.

The quantification of AMU at the farm-level represents an important step toward antibiotic stewardship as it provides detailed information on the quantity of antimicrobial use (AMU) at the level of end-user (farmer) and/or prescriber (veterinarian) (15). However, the estimation of quality on-farm AMU data remains challenging in many countries due to various factors such as poor animal health surveillance data, unavailability of treatment records, unauthorized use of antimicrobials, less awareness among farmers etc. (16–18).

To quantify the AMU at the farm level, various indices have been proposed (19). Some of the widely used metrics used are animal daily dose (ADD), antimicrobial drug use rate (ADUR), and used animal daily dose (UADD) (18, 20–22). The animal daily dose (ADD) in terms of grams/day for an animal can be obtained by multiplying the recommended “defined daily dose for animals” (DDD_kg_) of a drug for its main indication in a specified species by the approximate weight of an adult animal (23, 24). The number of animal daily doses (nADD) can be derived by dividing the total amount (mg or g) of medicine used by ADD, which is the product of actual animal weight and the standard dosage (24). The ADUR is equivalent to “daily doses per 1000 animal-days”, i.e., “nADD/1000 animal-days”, and is considered as a standardized measure for reporting ADD (6, 20, 25). ADUR is a time-sensitive measure which is not affected by the number of animals, and is useful in comparing AMU among the herds (21).

If the exact administered dose and the detailed data on the antibiotic application are known, the used daily dose (UDD), which is the administered dose per day per kilogram of a drug, can be calculated (25, 26). The UDD can be used to calculate the used animal daily dose (UADD) (in mg/day), which is the product of animal weight and UDD (mg/kg/day). The UADD can only be calculated from detailed data on antibiotic administration, and such metrics are considered as a representative of the actual field-level use of the drug, since the treatment duration, weight and number of diseased animals vary between the treatments (15, 24). Further, the number of used animal daily doses (nUADD) can be derived by dividing the total amount (mg or g) of medicine used by UADD, which is the product of actual animal weight and an estimate of the daily dose used for that antibiotic (22). As UDD represents more variations from the daily defined doses, the ratio of UDD and “defined daily doses for animals” (DDD_kg_) facilitates an estimate of deviation in the dose administered during treatment from the recommended dosage (27).

Though there are studies from developed nations on tracking antibiotic usage in farms, there are limited studies from India as well as from other developing nations on assessing AMU (20, 21, 28). Moreover, to date, there is little knowledge about the amount of HPCIAs used in the dairy sector of India (9). On-farm recording of AMU by employing the available methods like “bin method” and veterinary prescription records can measure the actual amount of antibiotics used on the farm (21). The earlier studies have observed the “bin method” as a suitable tool for monitoring AMU on the farm with better compliance than veterinary prescription records, particularly when the period of study is >6 months (29, 30). Further, the studies have depicted that AMU data from the “bin method” could be a suitable tool to measure antimicrobials administered by farmers and is efficient for detecting the practice of unauthorized use of antimicrobials (20, 21, 31).

The present study targets the state Punjab of India, which has the highest per capita milk availability and is one of the leading milk-producing state in the country (32). The continuous rise in the demand for milk in Punjab as well as from neighboring states generates demand-associated production pressure among the dairy farmers in the region. This has led to the shifting of the trend from household dairy herds to commercial-intensive dairy farming in the region. Therefore, it is important to assess the AMU in dairy herds of Punjab to observe a reflection on the quantity, frequency of administration, and types of antibiotics used in dairy animal production in the state. In light of this background, the objective of the present study was to evaluate the pattern and frequency of AMU among adult bovine animals using the “bin method” along with the treatment history records from Punjab dairy farms for a duration of 12 months.

## 2. Materials and methods

### 2.1. Herd enrolment

In the present study, forty-five farm owners were contacted through farm visits, and thirty-nine agreed to participate in the study. The farms were selected based on convenience and purposive sampling in order to include the farms from different geographical regions of Punjab. The farm owners were made clear about the purpose of the study and they provided their consent to use the antimicrobial usage (AMU) data of their farm for the study. The AMU data from the dairy farms were collected monthly from July 2020 until June 2021, i.e., for a period of 1 year. In the study, a total of 39 dairy farms [20 from household-level herds (those having 5 to 20 animals and mainly managed with manual labor by the family members) and 19 from commercial farms (those having more than 20 animals with semi- or fully-mechanized farm operations)] were selected to monitor the AMU pattern. However, one commercial farm refused to participate in the study after 3 months of enrolment, thereby the study was completed on 38 farms. The total adult bovine animal population in the selected 38 herds comprised of 1,010 animals including both cattle (*n* = 519) and buffaloes (*n* = 491). Further, heifers and calves were not included in the calculation of antibiotic mass as they represented only a minor share of the total AMU in the case of targeted dairy herds, moreover, the such population is frequently changing in the herds due to their regular sale or purchase procedures (33).

### 2.2. AMU data collection

The AMU data of the targeted herds were collected by placing forty-liter receptacles with round swing tops on the selected farms. The receptacles were placed at a convenient location on the farm and the farm owners as well as farmworkers were instructed to place the empty containers of all the drugs used for treatment in animals into these receptacles. Further, in the study region, concerned veterinarians, para-veterinarians and unauthorized practitioners were also requested to place empty drug packets in the placed bins. In case of an incomplete or one-time use of an antimicrobial (where the vial is not emptied), the treatment prescriptions were requested to place in the receptacles.

The receptacles were emptied from the participating farms at monthly intervals and the data were recorded about the product name, volume or weight used, concentration of the product, and the number of drug vials deposited in the receptacle. The information about the number and category of animals being treated (species, age, and approximate weight of the animal), the number of days treated, the route of administration, the information on the person administering medicines, and reasons for treatment were obtained from the farm owners at monthly intervals along with the empty vials of the used medicines.

### 2.3. Data analysis

All the data were entered in an Excel spreadsheet (Microsoft Corporation, Redmond, Washington, USA, 2016). The contents of the bin were quantified by calculating the total amount of antibiotics administered in weight (mg) of the active substance used in the animals. The frequency of the different used antibiotics (active substances) was calculated by accounting the empty vials deposited in the bin along with the treatment history records from the farm owners and/or farm workers at monthly intervals. The used metrics for AMU quantification were animal daily dose (ADD), antimicrobial drug use rate (ADUR) and used animal daily dose (UADD).

The animal daily dose (ADD) refers to the g/day dosage for an animal, and was obtained by multiplying the recommended average daily dose of the active pharmaceutical ingredient (DDD_kg_) by the approximate weight of an adult animal (20, 23, 24). The defined daily dose (DDD_kg_) designates the mg/kg/day dosage obtained from the DDD_vet_ calculations, which are the recommended value for each target species provided by the European Surveillance of Veterinary Antimicrobial Consumption (ESVAC)/ European Medicines Agency (EMA) (34, 35). In the case of products without prescribed DDD_vet_ measures, the on-label recommended dosage was used (36). Drugs with multiple antibiotic compounds such as the combination of sulfonamides with trimethoprim, amoxicillin-clavulanic acid/sulbactam/tazobactam, benzylpenicillin-benzathine, and benzylpenicillin-procaine were interpreted as single active substance (25). For the combination of trimethoprim-sulphonamides, DDD_vet_ was calculated for the minor substance, i.e., trimethoprim (37).

The ADD was calculated for each antibiotic administration by multiplying the recommended average daily dosage (DDD_kg_) for the antibiotic by the actual exposure weight (kg) of the treated animal (24). As the country-specific standard weights were not available and animal weights at treatment might differ substantially, the weight at treatment for each animal recorded in the present study was used for estimating the ADD. The parenteral antibiotic formulations were calculated per kilogram of animals with recorded individual weights of the animals at exposure. In the study, the median body weight of the adult dairy cow was found to be 400 kg (mean 421 kg, min 300 kg, max 520 kg) and the adult buffalo weight to be 500 kg (mean 525 kg, min 425 kg, max 650 kg). For intramammary products, the ADD for antibiotic “A”, was calculated using the formula: ADD = MG_DDDA_×U_DDDA_×F_DDDA_; where MG_DDDA_ is the dose (mg or IU) contained in a milliliter or an intramammary syringe of compound “A”, U_DDDA_ is the number of milliliters used in each administration, and F_DDDA_ is the number of times per day the compound is administered (38).

The recorded amount of antibiotic administered to the animal obtained from the collection bin during each treatment was then divided by the calculated ADD (the product of expected dosage and the average animal weight) to yield the nADD. These calculations were performed individually for each observation, and the nADD at the herd level was estimated for each antibiotic agent by adding all drug-specific nADDs (21).


$$\begin{array}{l} \text{nADD}=\frac{\text{Amount }\left(\text{in grams}\right)\text{ of active ingredient used in the treatment}}{\text{ADD }\left(\frac{\text{g}}{\text{day}}\right)}\\ =\frac{\text{Amount }\left(\text{in grams}\right)\text{ of active ingredient used in the treatment }}{\text{Recommended daily dosage }\left(\frac{\text{g}}{\text{kg}}\right)\text{ x Average animal weight }\left(\text{in kg}\right)\text{ at treatment}} \end{array}$$

Further, the herd level ADUR of various antibiotic groups was measured in the number of ADD/1000 animal-days using the formula described below (20, 21).


$$\begin{array}{l} \text{ADUR}\left(\text{ADD}/1000\text{ animal}-\text{days}\right)=\\ \frac{\text{Active ingredient used in the study period }\left(\text{g}\right)\text{ x }1000}{\text{ADD x Number of days in the study period x Number of animals at risk}} \end{array}$$

The amount of active substance(s) actually administered to the animal was calculated using the metric of UDD in mg/kg/day (25). The UDD for each antibiotic during each treatment was calculated separately for each data entry by dividing the actual amount of antibiotic compound administered (mg) by the number of animal times the average of the actual weight of the treated animals, and the treatment duration in days (39, 40). The used formula is:


$$\begin{array}{l} \text{UDD}\left(\frac{\text{mg}}{\text{kg x day}}\right)=\\ \frac{\text{Weight of active substance }\left(\text{mg}\right)}{\text{No}.\text{ of treated animals x Average weight }\left(\text{kg}\right)\text{ x treatment duration }(\text{days}}) \end{array}$$

The used animal daily dose or UADD was obtained from the product of animal weight and used daily dose (UDD). Further, the number of used animal daily doses or nUADD of each antibiotic was calculated as described by Flor et al. (22) by dividing the amount per antibiotic used by the UADD, which is the product of UDD and the animal weight at treatment (22). Similar to the nADD, the nUADD at the herd level was estimated for each antibiotic agent by adding all drug-specific nUADDs.


$$\begin{array}{l} \text{nUADD}=\frac{\text{Amount }\left(\text{in mg}\right)\text{ of active ingredient used in the treatment }}{\text{UADD}}\\ \;=\frac{\text{Amount }\left(\text{in mg}\right)\text{ of active ingredient used in the treatment }}{\text{UDD }\left(\frac{\text{mg}}{\text{kg}/\text{day}}\right)\text{ x Average animal weight }\left(\text{in kg}\right)\text{ at treatment}} \end{array}$$

Moreover, the UDD/DDD_kg_ ratios were also calculated to quantify the antibiotic consumption and correctness of the administered dosage, in which a ratio between 0.8 and 1.2 was considered as correct dosing. The under-dose and overdose were, respectively interpreted as values < 0.8 and >1.2 (25).

The descriptive statistics including unpaired *t*-test and graphical illustrations were carried out using Microsoft Excel 2016.

## 3. Results

### 3.1. Herd characteristics

The majority of farms (84.21%, *n* = 32) enrolled in the study were mixed-species dairy farms comprising of both cattle and buffaloes, whereas 5 were exclusively cattle farms and one was buffalo farm. Further, 52.63% (n=20) of the farms were house-hold level herds comprising < 20 animals, whereas 47.37% (*n* = 18) were commercial farms comprising more than 20 animals. In the selected herds, a total of 208 animal health related cases were reported in 1 year of study which required antibiotic use, of which mastitis was the most frequently reported disease condition in farms (50.48%, *n* = 105), followed by fever (20.67%, *n* = 43), reproductive problems (17.31%, *n* = 36), and diarrhea (4.32%, *n* = 9). Around 15 miscellaneous conditions were reported such as indigestion, inflammation, injury, skin infection, abscess, teat obstruction, edema, etc., each accounting for a negligible percentage of the total cases. Concerning the personnel administering regular treatment in farms, para-veterinarians were involved in treatment in 34.21% herds (*n* = 13), followed by “unauthorized practitioners” (frequently called “private doctors” in the villages) accounting for the treatment in 26.32% of the herds (*n* = 10). The farm owners themselves administered treatment in 23.68% of the herds (*n* = 9), where the 15.79% of herds (*n* = 6) were treated by veterinarians. However, dairy farmers consulted the veterinarians for the treatment of all the complicated cases (e.g., dystocia, recurring mastitis, severe injuries, fractures etc.) and the treatment failure cases attended by para-veterinarians/unauthorized practitioners/themselves.

### 3.2. Description of antimicrobial active ingredients

In the selected farms, a total of 265 commercial antibiotic products of 14 different antibiotics belonging to 9 groups were identified, of which the majority were injectable preparations. Out of the total antibiotic compounds administered, the highest number of antibiotic products were administered in the cases of mastitis (54.72%), followed by treatment of fever (19.62%), reproductive problems (15.47%), and diarrhea (3.40%). A total of 18 (6.79%) drugs were administered in case of various miscellaneous conditions like skin infection, abscess, indigestion, inflammation, injury, teat obstruction, oedema, etc.

**Figure 1:**
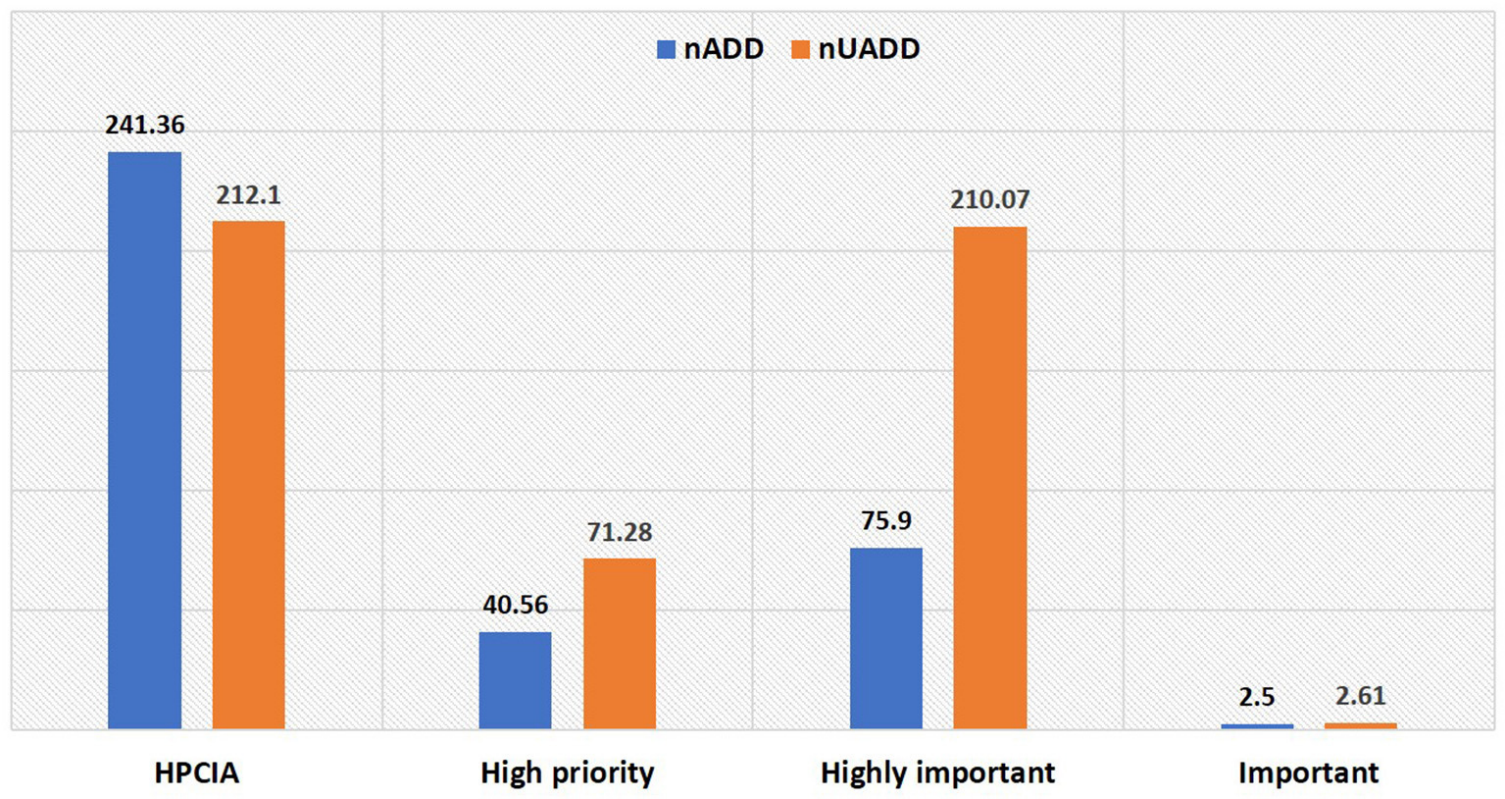
World Health Organization (WHO) Critically Important Antimicrobials (CIA) categorization of antimicrobials used in the herd in terms of number of used animal daily dose (nUADD) and number of animal daily dose (nADD).

The most common antibiotics used by frequency were enrofloxacin (89.47% herds; 21.51% products), followed by ceftriaxone (50% herds; 12.83% products), amoxicillin (50% herds; 12.83% products), oxytetracycline (55.26% herds; 11.70% products), and procaine penicillin (47.37% herds; 12.83% products) (Tables 1, 2). A total of 179 (67.55%) products administered contained antimicrobials of “critical importance” as per the WHO list (9) (Table 1, Figure 1). Of those, 125 (47.17%) products contained “highest priority critically important antimicrobials” (HPCIA) and 54 (20.37%) products contained “high priority critically important antimicrobials”. Of the total drugs administered in case of mastitis, the use of products containing “highest priority critically important antimicrobials” accounted for 52.42%, and “high priority critically important antimicrobials” comprised 26.21%. Similarly, “highest priority critically important antimicrobials” and “high priority critically important antimicrobials”, respectively accounted for 43.9 and 12.20% of total drugs used in reproductive problems, and 38.46 and 19.24% in case of fever (Table 1).

**Table 1:** Frequency of antimicrobial agents administered in the dairy herds (overall and condition wise).

**Group of antimicrobials**	**Name of antimicrobial**	**Overall drugs administered with the antimicrobial**		**Drugs administered in mastitis**		**Drugs administered in reproductive problems**		**Drugs administered in fever**	
		***n***	**%**	***n***	**%**	***n***	**%**	***n***	**%**
Cephalosporins (3rd Gen)	Ceftriaxone	34	12.83	25	17.24	4	9.76	3	5.77
	Ceftiofur	13	4.91	2	1.38	8	19.51	1	1.92
	Cefoperazone	12	4.53	10	6.90	–	–	–	–
	Ceftizoxime	7	2.64	6	4.14	–	–	–	–
Quinolones	Enrofloxacin	57	21.51	31	21.38	6	14.63	16	30.77
	Levofloxacin	2	0.75	2	1.38	–	–	–	–
Aminoglycosides	Gentamicin	18	6.79	12	8.28	–	–	5	9.62
Aminopenicillins	Amoxicillin	34	12.83	24	16.55	5	12.20	5	9.62
	Ampicillin	2	0.75	2	1.38	–	–	–	–
Tetracyclines	Oxytetracycline	31	11.70	7	4.83	4	9.76	18	34.62
Cephalosporins (1st Gen)	Cephalexin	9	3.40	–	–	9	21.95	–	–
Penicillins	Procaine penicillin	32	12.08	23	15.86	1	2.44	3	5.77
Sulphonamides	Sulphadiazine	10	3.77	1	0.69	–	–	1	1.92
Nitroimidazole	Metronidazole	4	1.51	–	–	4	9.76	–	–
Total		265		145		41		52	

**Table 2:** Frequency of herds using different antimicrobial groups in various conditions.

**Antimicrobial groups**	**Antimicrobial agent**	**Herds using antimicrobial agent (%)**	**Herds using antimicrobial agent in mastitis (%)**	**Herds using antimicrobial agent in reproductive problems (%)**	**Herds using antimicrobial agent in fever (%)**
Cephalosporins (3rd, 4th and 5th Gen.)	Ceftriaxone	19 (50)	15 (39.47)	4 (10.53)	3 (7.89)
	Ceftiofur	8 (21.05)	2 (5.26)	5 (13.16)	1 (2.63)
	Cefoperazone	10 (26.31)	8 (21.05)	–	–
	Ceftizoxime	6 (15.79)	5 (13.16)	–	–
Quinolones	Enrofloxacin	34 (89.47)	27 (71.05)	6 (15.79)	11 (28.95)
	Levofloxacin	2 (5.26)	–	1 (2.63)	–
Aminoglycosides	Gentamicin	13 (34.21)	9 (23.68)	–	5 (13.16)
Aminopenicillins	Amoxicillin	19 (50)	14 (36.84)	4 (10.53)	5 (13.16)
	Ampicillin	2 (5.26)	2 (5.26)	–	–
Tetracyclines	Oxytetracycline	21 (55.26)	6 (15.79)	3 (7.89)	14(36.84)
Cephalosporins (1st and 2nd Gen.)	Cefalexin	7 (18.42)	–	7 (18.42)	–
Penicillin	Procaine Benzylpenicillin	18 (47.37)	17(44.74)	1 (2.63)	3 (7.89)
Sulphonamides	Sulphonamides	10 (26.32)	2 (5.26)	–	1 (2.63)
Nitroimidazole	Metronidazole	4 (10.52)	–	4 (10.53)	–

### 3.3. Quantitative estimates of antimicrobial usage (AMU)

**Figure 2:**
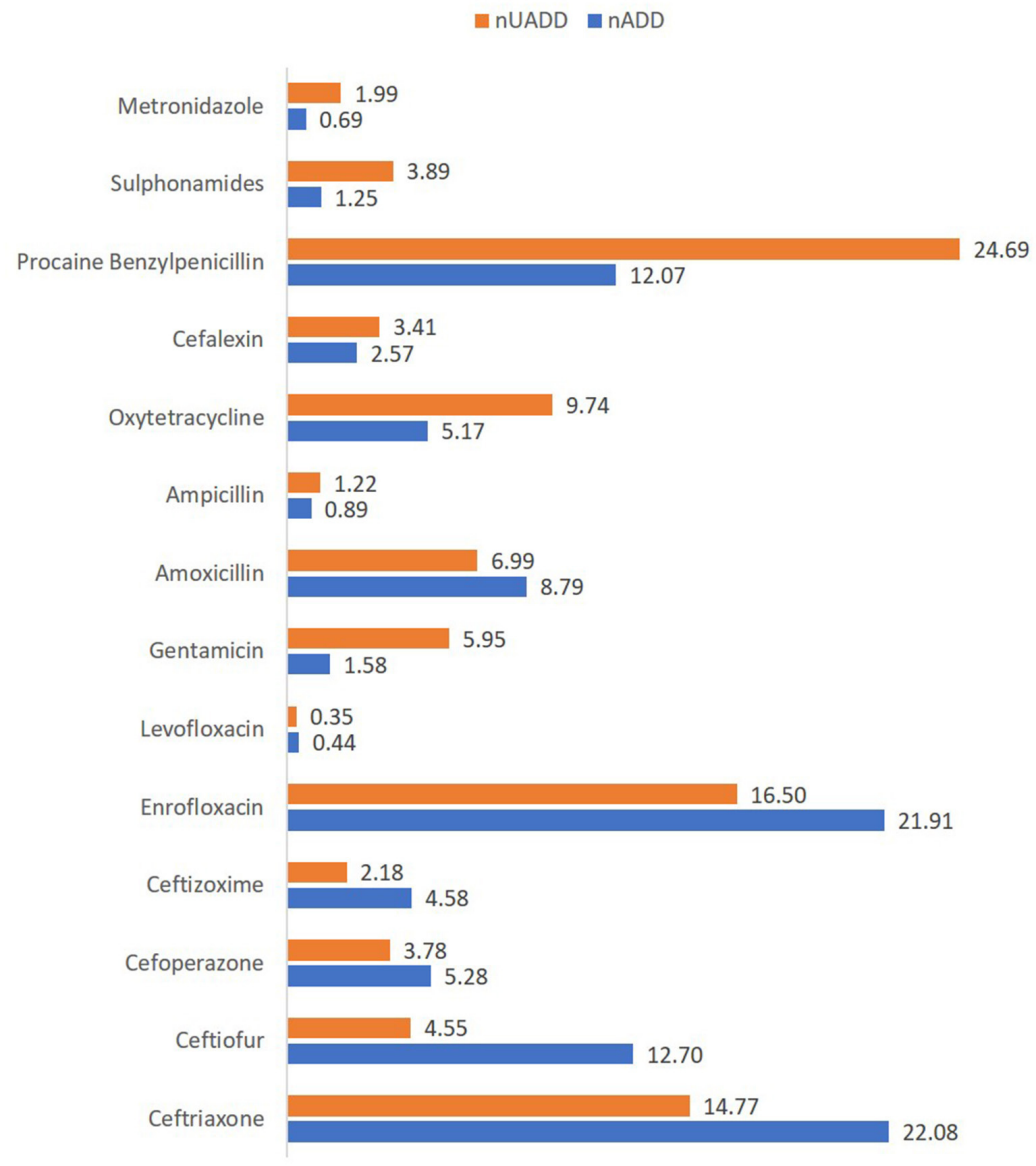
Percentage of the number of used animal daily dose (nUADD) and animal daily dose (nADD) of various antimicrobials used in the herds.

**Figure 3:**
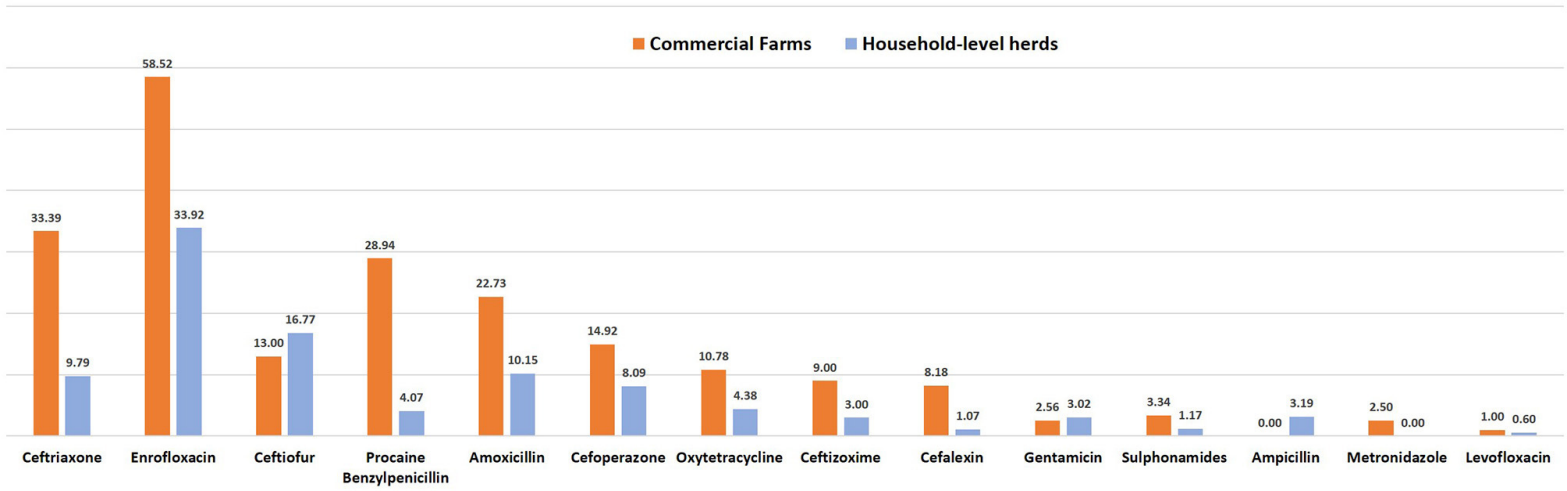
Antimicrobial usage (AMU) in terms of number of animal daily dose (nADD) in household level herds and commercial farms.

The AMU described in terms of nADD, ADUR, nUADD, along with the UDD/DDD_kg_ ratio of the used antibiotics in common disease conditions on the selected farms is provided in Tables 3, 4. On grouping antibiotic classes according to their prioritization by the WHO, penicillins followed by third-generation cephalosporins which belong to “HPCIA”, and aminopenicillins grouped under “high priority” were in use in the highest quantity in the herds (Figure 2). In the case of mastitis, penicillin (25.56%) followed by third-generation cephalosporins (22.02%) and aminopenicillins (20.01%) were the highest used in herds in terms of nADD. In the case of reproductive problems, 25.78% of the antibiotics used comprised of first-generation cephalosporins followed by aminopenicillins (20.13%), quinolones (15.28%), and third-generation cephalosporins (14.68%). In case of nADDs used in fever, oxytetracycline (34.44%) followed by enrofloxacin (20.79%) and aminopenicillin (12.15%) were largely used. Sulphonamides (96.34%) were the highest used in cases of diarrhea. In terms of overall nADD, the largest amount of antibiotic used in the herds was ceftriaxone, followed by enrofloxacin, ceftiofur and penicillin, respectively accounting for 22.08, 21.91, 12.70, and 12.07% of the total amount of antibiotics used (Figure 2). On comparing the household level and commercial farms, there was a significant difference in the antibiotic usage in terms of nADD in commercial farms (*p* < 0.007). The nADD used in household level and commercial farms along with different conditions in dairy herds is depicted in Figure 3 and Supplementary Figure 1. In the case of household level herds, enrofloxacin followed by ceftiofur and amoxicillin were largely used antibiotics, and for commercial dairy farms, enrofloxacin followed by ceftriaxone, procaine penicillin, and amoxicillin were mostly used antibiotics in terms of nADD. The nADD was highest for enrofloxacin in both house-hold level herds and commercial dairy farms, with an overall nADD of 33.92 for enrofloxacin in house-hold level herds and 58.52 in commercial dairy farms during the study period.

**Table 3:** Number of animal daily doses (nADD), animal daily dose (ADD) (g/day), number of used animal daily doses (nUADD), used daily doses (UDD) (mg/kg/day) and dosing ratio of antimicrobial compounds administered in the study herds.

**Antimicrobial group**	**Antimicrobial agent (active ingredient)**	**nADD in the herds (*n* = 38)**	**Median ADD (g/day)**	**nUADD in the herd (*n* = 38)**	**Median UDD (mg/kg/day)**	**UDD/DDD_kg_ Ratio**
Cephalosporins (3rd, 4th & 5th gen.)	Ceftriaxone	79.55	3.75	74.35	7.50	1.07
	Ceftiofur	45.77	0.50	22.89	1.25	2.00
	Cefoperazone	19.01	3.00	19.01	11.25	1.00
	Ceftizoxime	16.50	2.00	11.00	7.50	1.50
Quinolones	Enrofloxacin	78.93	2.10	83.08	5.00	0.95
	Levofloxacin	1.60	1.85	1.78	4.00	0.90
Aminoglycosides	Gentamicin	5.69	4.20	29.95	1.80	0.19
Aminopenicillins	Amoxicillin	31.68	4.15	35.20	7.50	0.90
	Ampicillin	3.19	4.88	6.13	4.50	0.52
Tetracyclines	Oxytetracycline	18.63	3.25	49.03	3.25	0.38
Cephalosporins (1st & 2nd Gen)	Cefalexin	9.26	2.80	17.15	6.75	0.54
Penicillin	Procaine Benzylpenicillin	43.50	5.20	124.29	3.25	0.35
Sulphonamides	Sulphonamides	4.51	5.20	19.61	3.00	0.23
Nitroimidazole	Metronidazole	2.50	2.00	10.00	1.25	0.25

**Table 4:** ADUR (i.e., ADD/1000 animal-days) of various groups of antimicrobials in the selected dairy herds.

**Antimicrobial group**	**Antimicrobial agent**	**nADD**	**ADUR**	**Median ADUR**
Cephalosporins (3rd, 4th and 5th Gen)	Ceftriaxone	79.55	0.39	0.010
	Ceftiofur	45.77	0.71	0.078
	Cefoperazone	19.01	0.20	0.021
	Ceftizoxime	16.5	0.25	0.023
Quinolones	Enrofloxacin	78.93	0.22	0.005
	Levofloxacin	1.6	0.09	0.046
Aminoglycosides	Gentamicin	5.69	0.05	0.003
Aminopenicillins	Amoxicillin	31.68	0.17	0.008
	Ampicillin	3.19	0.16	0.082
Tetracyclines	Oxytetracycline	18.63	0.09	0.004
Cephalosporins (1st & 2nd Gen)	Cefalexin	9.26	0.16	0.019
Penicillin	Procaine Benzylpenicillin	43.5	0.30	0.007
Sulphonamides	Sulphonamides	4.51	0.05	0.005
Nitroimidazole	Metronidazole	2.5	0.07	0.007

The antibiotic use by quantity measured as ADUR in terms of nADD for 1000 animal-days was highest for ceftiofur, followed by ceftriaxone, procaine benzylpenicillin, ceftizoxime, enrofloxacin, and cefoperazone (Table 4). In the present study, the lowest ADUR was reported for gentamicin, sulphonamides, and metronidazole. The highest median UDD (mg/kg/day) was observed for cefoperazone, followed equally by ceftriaxone, ceftizoxime, and amoxicillin. In terms of UADD, the highest amount of use was recorded for procaine penicillin (24.69%), followed by enrofloxacin (16.50%) and ceftriaxone (14.77%) (Table 3, Figure 2). In terms of overall nUADD, HPCIA such as third-generation cephalosporins and quinolones, respectively accounted for 25.27% and 16.86% of the total antibiotic use in the herds (Figure 2).

From the UDD/DDD_kg_ ratio, it was observed that penicillin, oxytetracycline, cephalexin, sulphadiazine, gentamicin, ampicillin, metronidazole, sulfadimethoxine and trimethoprim were frequently underdosed, while ceftizoxime and ceftiofur usually overdosed. The antibiotics such as enrofloxacin, ceftriaxone, amoxicillin, cefoperazone, and levofloxacin were usually dosed within the adequate dose range (i.e., UDD/DDD_kg_ =0.8–1.2).

## 4. Discussion

The need for robust monitoring systems for data collection and understanding the antimicrobial usage and consumption is crucial for addressing AMR in the animal husbandry sector as well as in humans, since many of the antibiotic classes are shared among both sectors (14, 34). In concordance with earlier studies (40, 42, 43), the present study involves analysis of annual data of AMU on dairy farms using bins for the collection of empty drug containers along with treatment history collected directly from the farm owners. In developing countries like India, many times antibiotic doses are not administered adhering to standard pharmacopeia for the recommended value for each target species, thereby, the on-farm quantification of antimicrobials can represent a more accurate measure for quantifying AMU (44).

Earlier studies have reported the use of “bin method” of AMU data collection to have good to excellent reliability for injectable and intramammary products, and is potentially preferable in countries like India where obtaining veterinary sales data is difficult (45). One of the advantages of the bin dataset is that “overreporting” of AMU is less likely as the record of only used empty vials is made from the bins (46). However, erroneous overreporting may occur if any subset of the animal population in which antibiotics are used is not taken into account, and the method is labor-intensive and utilizes many resources, making its routine application difficult. Farmers observed the bin method as convenient, however, there may be the chance of under-reporting if the researchers do not periodically collect the data from the farm or fail to motivate the farmers about placing the empty vails, or if any new worker joins the farm in between the study who is unaware of the ongoing study (28, 45). Theoretically, the treatment records are considered a precise method of measuring AMU in well-managed herds; however, the practical feasibility of this method requires constant commitment and effort from the people associated with the dairy farm, otherwise, it may result in incomplete data recording (47). Hence, the present study included the bin method along with the treatment history of data collection from farm workers to strengthen the study results.

The present study points toward the high use of critically important antimicrobials (CIAs) in animal production in study regions, where around 67.55% (179/265) administered products contained antimicrobials of “critical importance”, particularly for diseases such as mastitis, reproductive problems and fever in bovines. In accordance with an earlier study by Firth et al. (48), where the use of “HPCIA” in treatments of mastitis was reported to vary from 10–80%, the present study also reported 52.42% of the total drugs administered in mastitis to be under “HPCIA” category. Similarly, a study from Germany has reported that more than 32% of the antibiotics used during lactation were “HPCIA” (49). In the present study, cephalosporins, particularly third-generation cephalosporins made up 29.66% of antibiotic use in mastitis, 29.27% of use in reproductive problems, and 7.69% of use in fever. Similar to the present study, in Austrian dairy farms, 3rd and 4th generation cephalosporins were most frequently used, particularly for the treatment of mastitis and foot diseases (50), and 3rd generation cephalosporins accounted for 75% of intramammary antimicrobials used in the Wisconsin dairy farms during 2016–2017 (41). In a study on dairy farms in the United Kingdom, the use of highest priority, critically important antimicrobials (fluoroquinolones, third- and fourth-generation cephalosporins and colistin) was found to be predominant (45). An earlier study on veterinarians from India also reported the high usage of HPCIA such as quinolones (76.8%) and third-generation cephalosporins (47.8%) in dairy herds (51). Similarly, the present study also revealed higher use of quinolones and third-generation cephalosporins, both in terms of frequency and quantity of use. The high use of quinolones in India could have paved the increased resistance toward fluoroquinolones and cephalosporins among Gram-negative and Gram-positive bacteria in the country (52). Such AMU data at the regional level helps to identify the trends in antimicrobial usage and serves to inform health policy makers to initiate evidence-based responses to tackle this public health issue.

When quantifying antimicrobial use in animals, the choice of metric and denominators to use is complex, and numerous weight-based and dose-based metrics are widely used (19), and no single method is considered to be ideal in all situations (53). The present study has employed AMU quantification based on different metrics such as animal daily dose (ADD and nADD), antimicrobial drug use rate (ADUR), and used animal daily dose (UADD and nUADD). In line with the earlier studies where the AMU quantification from the same data set vary depending upon the metric calculated, the present study has also found variation in the AMU quantification in the data from the same herd, based on the standard dosage and the actual dosage, in terms of nADD and nUADD, respectively (24). Similar to the present study, deviations in the UDD and DDD_kg_ have been reported in previous studies (14, 22). The variation in the estimates of AMU can happen because of under- or overdosing by the treatment provider, or by using the standardized weight, since the animal weight at the time of treatment may be different from the standardized weight (22). In the present study, the daily dose metrics, the nADD and the nUADD, were calculated based on the specific (estimated) live weight of the animal at treatment instead of standardized weights which may be country- and livestock sector-specific, and the AMU calculation using more specific weights for the animals at exposure were found to be more precise (15, 24, 54).

The state Punjab of India is primarily an agrarian economy with dairying as an important source of income for farmers (32). With the increase in commercial dairy herds, an increase in antibiotic consumption is also expected in the region. The present study reports the higher use of antibiotics in commercial farms, particularly antibiotics such as enrofloxacin, third-generation cephalosporins like ceftriaxone, cefoperazone, ceftizoxime, tetracycline like oxytetracycline, benzylpenicillin etc. This increased antibiotic use in accordance with the scale of operation can be attributed to the direct marketing of veterinary antibiotics to farm owners and the stocking of antibiotics on the farm premises, particularly in the case of commercial farms (44, 55). There exists an efficient socio-economic basis of farmers which encourages their irrational antibiotic use, in which the ease of easy access to antibiotics, and the need for profits and fewer losses have caused an increase in non-prescription antibiotic consumption, many times compromising good husbandry practices (56).

In the present study, the farmers reported that in many cases the antibiotics were administered by unauthorized personnel such as para-veterinarians, unauthorized practitioners and farmers themselves. Earlier studies also have reported that antibiotic use in the dairy sector of India is predominated by para-veterinarians, unauthorized practitioners, and the dairy farmer themselves (51, 57). The predominance of informal practitioners was widely reported in the health systems of low- and middle-income countries, including India (58). In the present study, only 15.79% of the herds were observed to be primarily treated by veterinarians, highlighting the requirement for strengthening the veterinary services in the country. The treatment of the animals by unauthorized personnel (i.e., para-veterinarians, unauthorized practitioners and farmers themselves) in the targeted farms could explain the overdosage of certain antibiotics in the present study, particularly the higher generation cephalosporins such as ceftizoxime and ceftiofur, and underdosing of many antibiotics, like penicillin, oxytetracycline, gentamicin etc., which warrants immediate action for promoting judicious antibiotic usage. Apart from this, a multitude of other possible factors such as poor farm biosecurity, inadequate infection control practices along with the lack of compliance with regulatory frameworks could have resulted in the indiscriminate use of antibiotics in the targeted dairy herds.

The quantification of antibiotic usage is considered crucial to assess animal husbandry practices and the effectiveness of ongoing stewardship programs. Since the use of metrics based on actual dosage requires the measurement in terms of actual dose rate, the treatment duration and the weight of animals at exposure, as was available in this study, are costly and time-consuming, they may not always be feasible at the national level (23, 59). However, the detailed recording of AMU data as in the present study is recommended on sentinel farms, when feasible, to complement the national AMU data (60). The data of the present study can be further used to determine the associations between antibiotic usage and associated resistance, which can inform necessary improvements in the existing AMU/AMR surveillance programs. Such region-specific studies can guide the policymakers in the formulation of evidence-based stewardship and awareness programs among the stakeholders (e.g., veterinarians, para-veterinarians, farmers etc.).

## 5. Limitation

The antibiotics administered in most of the herds in the present study were by unauthorized practitioners, which might have led to inappropriate treatment duration in most cases, and thereby may have led to an under- or overestimation of the used daily doses, in comparison to the herds treated by the veterinarians. Further, we have tried to select the farms from various regions of Punjab, however, the inherent limitations of convenient and purposive sampling could have led to selection bias in the study. Moreover, antibiotic usage was calculated for herds considering only adult bovine animals, disregarding the contribution of other age categories in those herds, even though they are in minor proportion. In this context, further studies need to be performed to determine the contribution of calves and heifers to antimicrobial use in India.

## 6. Conclusion

The present study relied on farm-recording of antimicrobial usage (AMU) by using various metrics, i.e., animal daily dose (ADD), antimicrobial drug use rate (ADUR), and used animal daily dose (UADD). In the present study, around 67.55% of the products administered contained antimicrobials of “critical importance” as per the WHO list and of those, 47.17% of products contained ‘highest priority critically important antimicrobials' (HPCIA). The study also reports the deviation of the used daily doses from the recommended dosage of various antibiotics. These findings highlight the widespread use of HPCIA in treatment in the animal husbandry sector as well as the widely prevalent practice of animal treatment by unauthorized personnel, which necessitates prompt action from the government as well as the various stakeholders for prudent antibiotic usage in animal husbandry. Moreover, such epidemiological studies at a large scale are recommended to generate evidence-based data on AMU and related trends, which may provide insights to generate tailor-made strategies for curbing AMR at the regional and national levels.

## Data availability statement

The original contributions presented in the study are included in the article/Supplementary material, further inquiries can be directed to the corresponding author.

## Ethics statement

The individual permission of the farm owners was obtained for their voluntary participation in the study. The identity of the participants was kept confidential throughout the study. The Human Ethical Research Committee, Dayanand Medical College and Hospital, Ludhiana provided the necessary ethical approval to conduct the study under the Indian Council of Agricultural Research, Niche Area of Excellence project on Antibiotic Resistance: Animal-Human Interface (Ref. No. DMCH/R&D/2021/7).

## Author contributions

Conceptualization: DV, JB, PD, RS, JS, and JG. Data curation: DV and PD. Formal analysis, analysis, and original draft writing: DV, JB, and PD. Funding acquisition: JB, AA, and JG. Investigation: DV, JB, PD, RS, JS, and AA. Methodology and review and editing: DV, JB, PD, RS, JS, AA, and JG. Project administration: JB, AA, and JG. Supervision and validation: JB, PD, RS, JS, AA, and JG. All authors contributed to the article and approved the submitted version.
