# Supplementary material for: Monitoring of antimicrobial usage among adult bovines in dairy herds of Punjab, India: A quantitative analysis of pattern and frequency
Source: Front Vet Sci. 2023 Mar 30;10:1089307. doi: 10.3389/fvets.2023.1089307 (PMC10098197; doi:10.3389/fvets.2023.1089307)
Supplement: Supplementary file 1 [file Data_Sheet_1.docx]

**Supplementary Figure 1: Antimicrobial usage in terms of number of animal daily doses (nADD) in selected herds: (a) Overall antimicrobial usage (AMU) (b) AMU in mastitis (c) AMU in fever, (d) AMU in reproductive problems, (e) AMU in diarrhoea**

**(a)**

(b)

(c)

(d)

(e)
